# Sociodemographic predictors of and main reasons for COVID-19 vaccine hesitancy in eastern Oslo: a cross-sectional study

**DOI:** 10.1186/s12889-022-14261-y

**Published:** 2022-10-07

**Authors:** Lara Steinmetz

**Affiliations:** Faculty of Social Sciences, Oslo Metropolitan University, Oslo, Norway

**Keywords:** COVID-19, Vaccine hesitancy, Vaccine intention, Sociodemographic predictors of vaccine hesitancy, Reasons for vaccine hesitancy, Barriers to vaccine uptake

## Abstract

**Background:**

Vaccines are an essential public health strategy to curb viral infection spreading that hinge on vaccine uptake which may be threatened by vaccine hesitant individuals. This study aims to identify sociodemographic predictors of vaccine hesitancy, main reasons for vaccine hesitancy, and how these reasons are explained by sociodemographic characteristics during the COVID-19 pandemic.

**Methods:**

A cross-sectional study (*N* = 5 442) was carried out in June 2021. A web-based survey was conducted among six eastern districts in Oslo with high infection pressure. Sociodemographic variables included gender, age, country of birth, education, and household income. Binary logistic regression models were used to explore predictors of both vaccine hesitancy and specific reasons for hesitancy.

**Results:**

Vaccine hesitancy was low overall (5.8%). Findings indicate that participants with younger ages, lower education, and lower household income, and those born outside of Norway were prone to vaccine hesitancy. Over half of the vaccine hesitant sample cited barriers relating to confidence in the vaccines. Women and participants born in Norway were more likely hesitant due to fear of side effects and there being little experience with the vaccines. Otherwise, complacency barriers such as not feeling that they belonged to a risk group (46.1%), not needing the vaccines (39.1%), and wanting the body to develop natural immunity (29.3%) were frequently selected by participants.

**Conclusion:**

Different determinants of vaccine hesitancy among population groups demonstrate the need for clear public health communication about the risks, benefits, and importance of vaccines. Future studies with a larger sample should verify current findings and further explore the role of convenience barriers in health literacy and language. Health authorities should take these results into account and develop different public health strategies targeted at vulnerable population groups during the current and future pandemics to increase vaccine uptake and reach sufficient immunization.

**Supplementary Information:**

The online version contains supplementary material available at 10.1186/s12889-022-14261-y.

## Background

The COVID-19 pandemic, caused by severe acute respiratory syndrome coronavirus 2 (SARS-CoV-2), has killed an estimated 6 508 182 people worldwide since initial cases were identified in December 2019 in China [1]. In severe cases, the virus may cause complications such as acute respiratory failure, pneumonia, heart problems, organ failure, and death. The virus typically spreads between humans in close proximity and via droplets in oral, nasal or eye mucous membranes [2]. Vaccines are the foremost important strategy to fight this spread, but their effectiveness hinges on vaccine uptake, which may be greatly threatened by vaccine hesitancy [3–5]. Vaccine hesitancy is defined by the WHO as either delayed acceptance or refusal of a vaccine despite its availability. It has been identified as one of the top ten major global health threats that may undermine global efforts to control the viral spreading of disease and efforts to reach sufficient immunization, referred to as herd immunity [6, 7]. Vaccine hesitancy is however a complex problem as it continuously evolves across context, time, place, and different vaccines [4, 8].

Previous literature from varied countries has identified several sociodemographic factors contributing to vaccine hesitancy during the COVID-19 pandemic. A worldwide systematic review found women tend to be more vaccine hesitant than men [9], which is supported by the majority of literature [9–19]. As public health messages have emphasized the increased vulnerability for severe COVID-19 outcomes of older adults (especially 60+) and those with underlying health conditions compared to others [20, 21], vaccine hesitancy is mostly found among younger age groups [5, 11, 14–16, 19, 22–25]. Increased vaccine hesitancy is also found among ethnic minorities even though they have experienced disproportionate suffering from COVID-19 [9, 16, 17, 19, 22, 26–30]. Additionally, the majority of literature has found associations between vaccine hesitancy and low education [9, 11, 13–15, 17–19, 22–26, 31], as well as low income levels [9, 11, 16, 17, 19, 22–24]. Moreover, both low education and income are associated with low health literacy which may contribute to vaccine hesitancy. The majority of literature in other countries also found that people living in disadvantaged areas are more vaccine hesitant [9, 16, 18, 22, 31, 32]. Still, many inconsistent results across studies demonstrate that sociodemographic predictors of vaccine hesitancy cannot be assumed [8].

The WHO’s Strategic Advisory Group of Experts (SAGE) developed the “3Cs” model which categorizes reasons for vaccine hesitancy into three main types: confidence, complacency, and convenience [4, 7]. Confidence reasons refer to worries about the safety and effectiveness of the vaccine. Complacency encompasses individuals with low self-perceived risk of a vaccine-preventable disease where other life or health values weigh heftier [4]. High self-efficacy, where individuals may have high confidence in personal abilities of prevention and so view vaccines as unnecessary preventive measures, also creates complacency [33]. Convenience matters include availability, accessibility, affordability, and the widespread reach and understanding of health messages (health literacy) from authorities about the vaccine. Although COVID-19 vaccines have been actively offered to all Norwegian residents free of charge, convenience barriers resulting from low health literacy or language barriers cannot be excluded due to the high migrant density in eastern Oslo [7, 33, 34].

Due to the continuous evolution of vaccine hesitancy, research across and within countries on a subnational and regional level is crucial to identify predictors and address specific barriers in vaccine uptake, to identify vulnerable population groups, and to react deftly with more tailored and nuanced approaches [4, 9, 16, 17, 29]. The WHO’s SAGE has therefore long called upon governments’ responsibilities to address vaccine hesitancy ‘hotspots’ [4].

This study focuses on eastern Oslo, a generally disadvantaged area where vaccine hesitancy is suspected due to socioeconomic deprivation and high ethnic minority density [35, 36], even though vaccine acceptance is expected to be high in Norway in general due to high levels of trust in Norwegian authorities [32, 37–39]. In March 2021, several municipalities in Norway received extra vaccine doses due to consistent high COVID-19 infection rates [34, 35]. In Oslo, these were mostly assigned to the six eastern districts included in the study. At the time of this study, Pfizer, Moderna, AstraZeneca, and the Janssen vaccines were commonly accepted COVID-19 vaccines [40]. AstraZeneca and the Janssen vaccines were however suspended in Norway after March 2021 due to possible severe side effects [41, 42]. COVID-19 vaccines were offered to all Norwegian residents free of charge, but convenience barriers resulting from low health literacy or language barriers cannot be excluded due to the high migrant density in eastern Oslo [7, 33, 34].

Undoubtedly, vaccine development is necessary for combatting a pandemic, but it is not sufficient. If enough people remain vaccine hesitant, herd immunity cannot be reached and detrimental health consequences will follow, demonstrating an urgent need for social research. This study contributes to the literature in several ways. First, by focusing on Norway, it adds to the body of information on vaccine hesitancy in countries that are underrepresented in such research [24]. Further, as current literature has primarily focused on measuring hypothetical vaccine intention, few studies have assessed vaccine hesitancy after the arrival of the COVID-19 vaccines. By using survey data collected during the COVID-19 pandemic from a potential ‘hotspot’ of six prioritized districts, this study addresses how vaccine hesitancy is predicted by sociodemographic characteristics. This approach enables the identification of population groups who may be vulnerable to disproportionate suffering, as well as the main reasons for vaccine hesitancy in these groups. Insights into these reasons and comparison of their predictors will aid the development of prioritization strategies and the tailoring of targeted public health responses to effectively reach hesitant subgroups and increase vaccine uptake [9, 15, 26, 29, 31, 43, 44]. Particularly during a pandemic, vaccine hesitancy is a concern for all as it is crucial for life and death and the burden of this behavior can have a worldwide impact. This study therefore has an immediate global implication both in fighting the current pandemic, as well as preparedness for ones to come.

## Methods

This cross-sectional study uses primary data from a survey database developed by researchers at OsloMet’s Centre for Research on Pandemics & Society (PANSOC) and the Pandemic Centre at the University of Bergen. The survey was administered by an external firm, Kantar, and conducted online in June 2021 among residents in the six eastern districts of Oslo (Alna, Bjerke, Gamle Oslo, Grorud, Søndre Nordstrand, and Stovner) that were prioritized for vaccination. These areas were also chosen due to high migrant density as one goal of the overall project was to investigate disparities based on migrant status. Therefore, the survey was translated into six different languages (English, Somali, Arabic, Urdu, Polish, and Norwegian) to encourage participation among ethnic minorities.

The sample was recruited proportionately according to the population in the different districts. With access to all phone numbers from a population database, 59 978 texts were sent out to residents above 18 years. Response rates were low (9.1%), and 5 447 participants completed the survey. Six respondents were excluded during the data cleaning process due to rapid survey completion, unclarity of answers to open-ended questions, or a large number of skipped questions. This resulted in a total sample of 5 442 respondents.

Collected sociodemographic characteristics included gender, age, education level, household income, and whether the respondent was born in Norway (Table1).

Vaccine hesitancy is considered in this study according to the WHO definition, which measures vaccine hesitancy based on vaccine intention. Hesitant individuals are people who show no intention or are unsure about taking the vaccine [4]. In the survey, participants were asked “If you have not yet, are you going to get vaccinated against COVID-19?”, with ‘Yes’, ‘No’, and ‘Unsure’ as response options. Participants who answered ‘No’ or ‘Unsure’ are classified as vaccine hesitant (Table2). Vaccine hesitant participants were subsequently asked to choose reasons for their hesitancy (Table3). Participants could select multiple reasons from the 16 possible options, which were coded as binary variables. Reasons chosen by more than 15% of the hesitant sample were considered as main reasons for further analyses. All survey questions included in this study can be found in Additional File 1.

### Statistical analyses

There were three main goals of the analyses: (1) to explore whether and how sociodemographic characteristics predicted vaccine hesitancy, (2) to identify the most frequently reported reasons for vaccine hesitancy, and (3) to assess how sociodemographic characteristics predicted those main reasons. As all variables were categorical, with binary outcome variables, logistic regression models were employed to predict odds ratios for goals 1 and 3. Unanswered questions were excluded from analyses, which resulted in sometimes large numbers of missing cases. Most of these blank answers were for questions on income (*n* = 876), education (*n* = 54), or country of birth (*n* = 35). No concerns for multicollinearity among the variables were indicated as no VIF-values exceeded 1.2. Statistical analyses were conducted in IBM SPSS Statistics 27.0 (Armonk, NY: IBM Corp), using an alpha level of 0.05.

## Results

### Sociodemographic characteristics

Of the 5 442 participants, nearly 60% were women (Table1). Participants aged between 18 and 29 years constituted 13.5% of the sample, while the other age groups (30–44, 45–59, 60+) each comprised approximately 30%. The majority of the sample was born in Norway (78.6%) and most participants completed a university/college degree equivalent to 4 years (31.3%) or over 4 years (28.5%). The median household income category was 800 000-999 999 Norwegian kroner (NOK). Data comparison between Norway’s statistical office and the survey indicated overrepresentations of women, people of older age, participants born in Norway, and people with higher education (university) [45].

**Table 1:** Sociodemographic characteristics of participants and representativeness (*N =* 5442)

Sociodemographic characteristics	Sample composition	Population composition
	***n*** **(%)**	***n*** **(%)**
**Gender**		
Male	2 270 (41.7)	122 776 (50.5)
Female	3 171 (58.3)	120 511 (49.5)
**Age**		
18–29	737 (13.5)	41 292 (21.4)
30–44	1 637(30.1)	63 981 (33.2)
45–59	1 496 (27.5)	45 715 (23.8)
60+	1 571 (28.9)	41 636 (21.6)
**Born in Norway**		
Yes	3 553 (78.6)	121 081 (49.8)
No	966 (21.4)	122 206 (50.2)
**Highest completed education**		
Primary (10, 7 year)	329 (6.0)	55 848 (28.9)
Higher general	785 (14.4)	60 209 (31.1)
Higher vocational	512 (9.4)	
Vocational school/vocational education (1/2–2 years) based on upper secondary vocational education	515 (9.5)	
University < = 4 years	1 702 (31.3)	49 994 (25.9)
University > 4	1 550 (28.5)	27 347 (14.1)
**Gross household annual income**		
Under 200.000 kroner	142 (3.1)	
200000-399999 kroner	435 (9.6)	
400000-599999 kroner	797 (17.6)	
600000-799999 kroner	742 (16.4)	
800000-999999 kroner	667 (14.8)	
1000000-1199000 kroner	626 (13.9)	
1200000-1399000 kroner	469 (10.4)	
1400000 kroner or more	641 (14.2)	

**Notes.** *Population and sample frequencies visualize the representation of the six prioritized districts (Alna, Gamle Oslo, Bjerke, Grorud, Søndre Nordstrand and Stovner)

*Due to different categories at Norway’s statistical office (“Statistikkbanken”), the education variable in the survey was collapsed to match and compare categories

*Only average income per districts was available at Norway’s statistical office. Therefore, no comparison with the survey categories could be made

### Vaccine distribution, uptake, and intention

The majority of participants (77.1%) had received a vaccine offer of whom 87.1% (*n =* 3 657) had taken the vaccine (Table2). Participants who had not yet received an offer for the vaccine (*n =* 1244) and those who had gotten an offer, but declined this (*n =* 541) were asked about their vaccine intention (“Will you take the vaccine?”) (*n =* 1 785). Of those who were asked this question, 104 (5.8%) answered ‘no’ and 212 (11.9%) answered ‘unsure’. The 316 vaccine hesitant individuals thus constitute 5.8% of the total sample. Additional File 1 further visualizes the flow of the survey questions.

**Table 2:** COVID-19 vaccine offers, recipients, and intention

	*n* (%)
**Received COVID-19 vaccine offer**	
Yes	4 198 (77.1)
No	1 244 (22.9)
Total	5 442 (100)
**Taken COVID-19 vaccine**	
Yes	3 657 (87.1)
No	541 (12.9)
Total	4 198 (100)
**Will take COVID-19 vaccine**	
Yes	1 469 (82.3)
No	104 (5.8)
Uncertain	212 (11.9)
Total	1 785 (100)

### Sociodemographic predictors of vaccine hesitancy

**Fig. 1:**
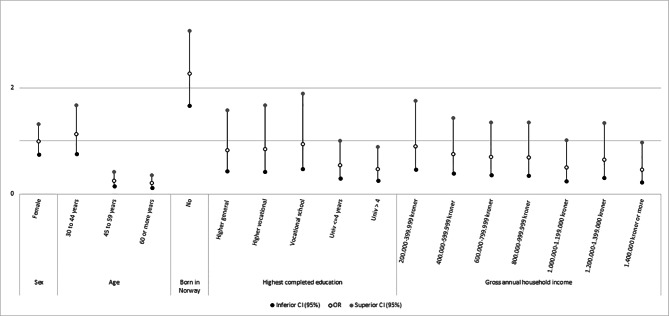
Logistic regression predicting vaccine hesitancy by sociodemographic predictors (*n* = 4518). (**Note.** Reference groups: Male, Age 19–29, Born in Norway, Primary education, Under 200 000 kroner)

Age differences in hesitancy were observed where those aged above 45 years had significantly lower odds ratios (i.e., lower likelihood of hesitancy) compared to those aged 18–29 (Fig.1). Further, participants born outside of Norway were more likely to be vaccine hesitant than those born in the country. Individuals with more than 4 years of university education were nearly 50% less likely to be hesitant compared to those completing primary education. Participants in the highest income group were also less likely to be hesitant compared to the lowest income group.

### Reasons for vaccine hesitancy

In the hesitant subsample, more than half feared the risk of side effects (55.8%) or thought there was too little experience with the use of the vaccine (50.2%) (Table3). Otherwise, hesitant individuals did not feel like they belonged to a risk group for severe COVID-19 disease (46.1%), did not feel the need for the vaccine (39.1%), or wanted their body to develop natural immunity (29.3%). Apart from the “Other” response (19.9%), no other reasons were selected by more than 15% of the subsample.

**Table 3:** Main reasons for vaccine hesitancy (*n =* 317)

Reasons for vaccine hesitancy	Yes	No
	***n*** **(%)**	
Risk of side effects from the COVID-19 vaccines	177 (55.8)	140 (44.2)
There is too little experience with the use of the vaccines	159 (50.2)	158 (49.8)
I do not belong to any of the risk groups for severe COVID-19 disease	146 (46.1)	171 (53.9)
Do not need the vaccine: rarely/never sick, not in the target group, I tolerate the flu well and probably COVID-19 also	124 (39.1)	193 (60.9)
I want my body to develop natural immunity	93 (29.3)	224 (70.7)
Other	63 (19.9)	254 (80.1)
I do not trust the recommendations from the health authorities/municipality	46 (14.5)	271 (85.5)
I do not think that the COVID-19 vaccines work	44 (13.9)	273 (86.1)
There is little infection in society	35 (11.0)	282 (89.0)
General objections to vaccines: I am vaccine opponent, never taken vaccines, in principle, not comfortable with new vaccines, heard a lot of strange things, etc.	22 (6.9)	295 (93.1)
Media attention	22 (6.9)	295 (93.1)
Afraid of/do not like doctors/syringes	21 (6.6)	296 (93.4)
Do not need to protect myself	17 (5.4)	300 (94.6)
Do not need to protect family/community	13 (4.1)	304 (95.9)
I do not trust healthcare professionals	8 (2.5)	309 (97.5)
Religious reasons	4 (1.3)	313 (98.7)

**Note**. One inconsistent respondent was not captured as hesitant in the variable on vaccine intention (*n = 316)* but was included in the variable on reasons against the COVID-19 vaccine. Hence, *n = 317*. This inconsistency did not influence any further results and was thus kept in the analysis

### Sociodemographic predictors of the main reasons for vaccine hesitancy

Table4 summarizes the results of the logistic regression models with each of the most frequent main reasons as outcomes. The risk of side effects reasoning shows a significantly higher odds ratio for women vs. men, and a lower odds ratio for participants born outside of Norway. Women also reported higher likelihood than men due to concerns about lack of experience with the vaccines, as did participants aged between 30 and 44 years compared to those aged 18–29. However, participants born outside Norway were less likely to report hesitancy for this reason compared to those born in Norway. For the reason of not feeling like they belonged to a risk group, respondents older than 60 years had a significantly and markedly lower odds ratio compared to the 18–29 reference group, and those born outside Norway also were less likely to be hesitant for this reason. Income also was significant in this model; the highest income group (> 1 400 000 NOK or more) and those in the 400 000-599 999 NOK group both hadhigher odds ratios compared to the reference of the lowest income group (< 200 000 NOK). Similarly, age and income were also significant predictors of the reason of feeling no need for the vaccine. Participants aged over 60 years reported low odds ratios for this reason. Compared to the lowest income group, the two highest income groups and the 400 000-599 999 NOK groups had higher odds ratios. Vaccine hesitancy due to the desire to naturally develop immunity was not significantly predicted by any of the sociodemographic characteristics. Overall, while the specific patterns varied, the most relevant variables associated with different reasons for vaccine hesitancy appear to be female gender, older age, being born in the country, and higher income.

**Table 4:** Logistic regression predicting the likelihood of vaccine hesitancy due to main reasons based on sociodemographic predictors (N = 210)

Predictor variables	Risk of side effects	Too little experience	Not in risk group	No need	Natural immunity
	OR (95% CI)	OR (95% CI)	OR (95% CI)	OR (95% CI)	OR (95% CI)
**Gender**					
Male	1.00 (Ref)	1.00 (Ref)	1.00 (Ref)	1.00 (Ref)	1.00 (Ref)
Female	1.99 (1.07-3.70)*	1.88 (1.02-3.46)*	1.09 (0.59-2.01)	0.78 (0.42-1.45)	0.67 (0.35-1.26)
**Age**					
18-29	1.00 (Ref)	1.00 (Ref)	1.00 (Ref)	1.00 (Ref)	1.00 (Ref)
30-44	1.83 (0.80-4.16)	2.70 (1.16-6.25)*	0.64 (0.28-1.45)	0.62 (0.27-1.43)	1.59 (0.65-3.89)
45-59	0.88 (0.30-2.54)	2.54 (0.85-7.65)	0.42 (0.14-1.21)	0.39 (0.13-1.20)	1.26 (0.39-4.00)
60+	1.21 (0.37-3.92)	1.14 (0.36-3.62)	0.05 (0.01-0.26)***	0.29 (0.08-0.97)*	1.20 (0.34-4.20)
**Born in Norway**					
Yes	1.00 (Ref)	1.00 (Ref)	1.00 (Ref)	1.00 (Ref)	1.00 (Ref)
No	0.45 (0.23-0.87)*	0.33 (0.17-0.64)**	0.65 (0.34-1.24)**	0.56 (0.29-1.09)	1.01 (0.51-2.01)
**Highest completed education**					
Primary (10, 7 year)	1.00 (Ref)	1.00 (Ref)	1.00 (Ref)	1.00 (Ref)	1.00 (Ref)
Higher general	3.56 (0.90-14.06)	1.39 (0.36-5.34)	0.94 (0.22-4.04)	2.35 (0.51-10.89)	0.82 (0.22-3.08)
Higher vocational	1.31 (0.31-5.59)	0.45 (0.10-2.03)	0.97 (0.20-4.67)	1.65 (0.32-8.44)	0.45 (0.10-1.97)
Vocational school/vocational education (1/2-2 years) based on upper secondary vocational education	1.70 (0.40-7.26)	1.40 (0.32-6.13)	0.72 (0.15-3.39)	2.64 (0.52-13.29)	0.52 (0.12-2.23)
University <= 4 years	1.51 (0.40-5.64)	0.88 (0.23-3.32)	1.27 (0.30-5.39)	1.35 (0.30-6.12)	0.42 (0.11-1.62)
University > 4	1.34 (0.35-5.08)	1.49 (0.39-5.77)	1.19 (0.28-5.07)	1.31 (0.28-6.03)	0.31 (0.08-1.26)
**Gross annual household income**					
Under 200000 kroner	1.00 (Ref)	1.00 (Ref)	1.00 (Ref)	1.00 (Ref)	1.00 (Ref)
200000-399999 kroner	3.09 (0.75-12.70)	0.97 (0.24-3.86)	2.70 (0.54-13.52)	4.79 (0.82-27.89)	0.62 (0.14-2.66)
400000-599999 kroner	1.59 (0.42-6.03)	1.20 (0.32-4.56)	5.80 (1.26-26.68)*	11.60 (2.09-64.42)**	1.58 (0.42-6.00)
600000-799999 kroner	3.73 (0.91-15.36)	1.57 (0.40-6.16)	4.10 (0.87-19.32)	4.34 (0.76-24.79)	1.03 (0.26-4.12)
800000-999999 kroner	2.85 (0.69-11.78)	1.70 (0.42-6.89)	4.30 (0.90-20.63)	4.34 (0.75-25.21)	0.58 (0.13-2.58)
1000000-1199000 kroner	3.48 (0.73-16.53)	1.96 (0.42-9.18)	4.82 (0.91-25.68)	2.66 (0.40-17.68)	0.99 (0.20-4.79)
1200000-1399000 kroner	2.24 (0.49-10.33)	1.28 (0.28-5.82)	4.01 (0.75-21.37)	7.77 (1.23-49.27)*	0.64 (0.13-3.29)
1400000 kroner or more	3.06 (0.61-15.30)	1.53 (0.31-7.57)	16.58 (2.58-106.78)**	32.90 (4.43-244.16)**	3.02 (0.61-14.91)
**Nagelkerke R2**	0.162	0.196	0.221	0.218	0.101

Notes. *p < .05; ** p < .01; *** p < .001

## Discussion

Several sociodemographic predictors of vaccine hesitancy were identified. Increasing age (45+) predicted lower likelihood of vaccine hesitancy compared to younger participants. In contrast, using country of birth as indicator for ethnic minorities, participants born outside of Norway reported higher likelihood of vaccine hesitancy compared to those born in Norway. These results align with an abundance of literature [5, 9, 11, 14–19, 22, 26, 30, 43], and are particularly relevant since both older age groups and ethnic minorities have increased vulnerability to severe COVID-19 outcomes [20, 21, 27, 28]. Whilst the results suggest appropriate risk perception among older adults regarding their vulnerable position, the opposite is the case for those born out of the country, whose vaccine hesitancy likely increases their vulnerability. Further, higher education (university) and high household income (> 1 400 000 NOK) predicted lower likelihood of vaccine hesitancy compared to lower education (primary) and low household income (< 200 000 NOK). These results also are consistent with the majority of literature that found a tendency towards hesitancy among those with lower education and income [9, 11, 13–15, 17–19, 22–24, 26, 31]. No significant gender differences in COVID-19 vaccine hesitancy were found.

Vaccine hesitancy was mainly due to confidence and complacency concerns as grouped by the “3Cs” model [4, 7]. Findings suggest that barriers relating to convenience were successfully reduced as the vaccines were made accessible by actively offering the COVID-19 vaccines to all Norwegian residents without costs. Confidence concerns were predominantly grounded in the fear of side effects and little experience with the vaccines, which is likely related to the fact that data collection took place not long after the introduction of the rapidly developed COVID-19 vaccines [46]. With regard to complacency reasons, participants mostly did not feel like they belonged to a risk group, felt no need for the vaccine, and/or wanted their body to develop natural immunity. These responses may be due to a lack of evidence at the time of how long immunity following infection persists and the risk of re-infection. Few participants stated that they had no need to protect themselves, family, and their communities which aligns with the low observed hesitancy rates. Further, results showed little distrust in health authorities and professionals, which is consistent with observed high trust in Norwegian authorities compared to other nations [32, 37–39]. The suspension of vaccines with higher risk of side effects from Norway’s immunization programme may have contributed to this trust, although fear of side effects still was an important barrier. Still, in an area where vaccine hesitancy was expected to be high, observed rates of hesitancy were nearly twice as low as was anticipated in Norway [32], which may indicate an underestimation of vaccine hesitancy. Finally, hesitancy due to religious reasons was the most infrequently selected option. As Oslo has the highest density of religious groups in Norway [47], this was unanticipated and may reflect an underrepresentation of ethnic minorities.

Findings show that women are more likely hesitant than men due to confidence barriers such as the risk of side effects and little experience with the COVID-19 vaccines. While participants born outside of Norway were more likely to be vaccine hesitant than those born in the country, they were less likely to be significantly associated with confidence-related reasons. Convenience barriers among ethnic minorities, such as whether health information is misunderstood or not accessed possibly due to barriers in health literacy or language, may play a larger role [7, 33–35]; further research is needed to confirm this speculation. Complacency barriers were less reported among older age groups (45+), participants born outside of Norway and in both lower (400 000-599 999 NOK) and higher household incomes (more than 1 200 000 NOK). These results further suggest successful public health communication and good health literacy among participants concerning age-related risk factors of COVID-19. Due to the high number of missing values for income, the reliability of results is questionable, which may explain why there were no significant findings for this variable. Although education significantly predicted vaccine hesitancy likelihood, no significant relations were found with any of the main reasons. This may also denote convenience barriers linked to health literacy as education is identified as a robust predictor of vaccine hesitancy [9, 11, 13–15, 17–19, 22–24, 26, 31]. This too calls upon the need for more research to confirm this speculation.

### Strengths and limitations

To my knowledge, this is the first study that addresses vaccine hesitancy in Norway after the arrival of the COVID-19 vaccines. It addresses vaccine hesitancy in an anticipated hotspot of a country that is underrepresented in literature, thus providing an important contribution to empirical literature. As such, in-depth insights into vaccine hesitancy are provided in which behavioural trends may be discovered which will be crucial in pandemic preparedness. Moreover, findings are linked to theoretical frameworks to increase relevance for forthcoming pandemics. There are also some limitations to this study. Statistical power and generalizability of the regression models were less strong than desired due to the small sample size and missing cases on the income, education, and/or country of birth variables. Furthermore, possible underrepresentation of several subgroups may have resulted in a non-response bias. Although this problematizes assessment of vaccine hesitancy, associations between variables seem rather insensitive to these concerns. It can however be argued that even with high nonresponse rates, results are not necessarily biased and are still scientifically valuable [48]. Lastly, the focus on the six eastern districts in Oslo limits the ability to compare to other districts or areas in Norway, while the studies’ cross-sectional design prevents causal inferences [49].

### Implications

Future research should aim to integrate both theoretical frameworks and empirical data on vaccine hesitancy to develop useful strategies for pandemic preparedness. Further efforts should also explore the role of convenience barriers in health literacy and language among ethnic minorities and those with lower education. Moreover, different barrier trends suggest the need for tailored policy strategies targeted to vulnerable subgroups to increase vaccine willingness. As results indicated high trust in health authorities and professionals, clear health communication about the risks, benefits, and importance of the vaccines should be provided to improve health literacy and vaccine willingness. Similar research should be carried out on a larger sample to produce more robust analyses.

## Conclusion

Results highlight the vulnerability of younger age groups, ethnic minorities, and those with lower education and lower household income to COVID-19 vaccine hesitancy. Different trends among subgroups emphasize the importance of clear public health communication about the risks, benefits and importance of vaccines. Norwegian health authorities should consider these findings when developing tailored strategies targeted at hesitant subgroups during the COVID-19 pandemic to increase vaccine uptake and reach sufficient immunization.

## Electronic supplementary material

Below is the link to the electronic supplementary material.

Supplementary Material 1

## Data Availability

The datasets generated and/or analysed during the current study are not publicly available due to wishes of an OsloMet Centre of Research; the Centre for Research on Pandemics and Society (PANSOC), but are available from the corresponding author on reasonable request.

## List of abbreviations

Norwegian kroner: NOK.
Strategic Advisory Group of Experts: SAGE.
World Health Organization: WHO.
