# Supplementary material for: Sociodemographic predictors of and main reasons for COVID-19 vaccine hesitancy in eastern Oslo: a cross-sectional study
Source: BMC Public Health. 2022 Oct 7;22:1878. doi: 10.1186/s12889-022-14261-y (PMC9542469; doi:10.1186/s12889-022-14261-y)
Supplement: Supplementary file 1 — Supplementary Material 1 [file 12889_2022_14261_MOESM1_ESM.pdf]

Overview survey questions

Below are all included survey questions that were included in this study. Since the survey was conducted in Norway, the original survey questions are formulated in Norwegian, but have also been translated to English. To enhance transparency of the questionnaire, both are displayed.

The codes of the questions are also included. Q0… stands for 'Question', followed by the number of the question in the questionnaire. The survey flow depends on the answer of the participant. See the figure below for the flow of questions in the survey. The green arrows indicate where vaccine hesitancy is discovered.

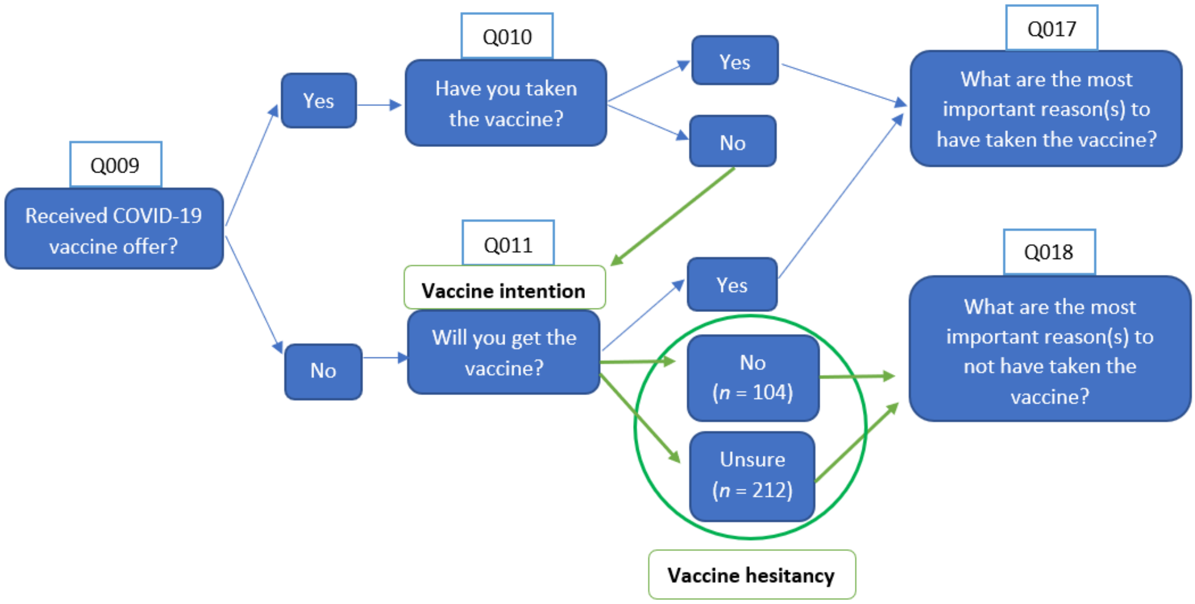

Q009 - Tilbudvaksine: Fått tilbud om vaksine

Har du fått tilbud om vaksine mot COVID-19?

Have you been offered a vaccine against COVID-19?

1 - Ja – *yes*

2 - Nei - *no*

Q010 - Vaksinertcovid: Har vaksinert seg mot Covid-19

Har du vaksinert deg mot COVID-19?

Have you been vaccinated against COVID-19?

1 - Ja – *yes*

2 - Nei – *no*

Q011 - Kommercovid: Kommer til å ta vaksinen

Kommer du til å ta vaksine mot COVID-19?

If you have not yet, are you going to get vaccinated against COVID-19?

1 - Ja – *yes*

2 - Nei – *no*

3 - Usikker – *unsure*

Q018 - Viktigstikkevaksinen: Viktigste grunner til ikke å ha tatt vaksinen

Hva er viktigste grunner til at du ikke har tatt eller vil ta, eller er usikker på om du vil ta koronavaksinen?

What are the most important reasons that you have not taken-, or want to take-, or are unsure whether you want to take the COVID-19 vaccine?

1 - Jeg tilhører ikke en av risikogruppene for alvorlig COVID-19 sykdom

- I do not belong to any of the risk groups for severe COVID-19 disease

2 - Har ikke behov for vaksine: Sjelden/aldri syk, ikke i målgruppen, tåler en influensa og sannsynligvis også COVID-19

- Do not need the vaccine: Rarely / never sick, not in the target group, I tolerate the flu and probably COVID-19 also

3 - Religiøse grunner

- Religious reasons

4 - Det er ikke mye smitte i samfunnet

- There is not much infection in the community

5 - Har ikke behov for å beskytte meg selv

- Do not need to protect myself

6 - Har ikke behov for å beskytte familie/samfunn

- Do not need to protect family / community

7 - Redd for/liker ikke leger/sprøyter

- Afraid of / do not like doctors / syringes

8 - Ønsker at kroppen skal utvikle naturlig immunitet

- Want the body to develop natural immunity

9 - Tror ikke koronavaksinene virker

- Do not think the COVID-19 vaccines work

10 - Risiko for bivirkninger av koronavaksinene

- Risk of adverse reactions to COVID-19 vaccines

11 - Det er for lite erfaring med bruk av vaksinene

- *There is too little experience with the use of the vaccine*

12 - Generelle motforestillinger mot vaksine: jeg er vaksinemotstander, aldri tatt vaksiner, prinsipielt, ikke komfortabel med nye vaksiner, hørt mye rart, etc.

- *General objections to vaccines: I am a vaccine opponent, never taken vaccines, in principle, not comfortable with new vaccines, heard a lot of strange things, etc.*

13 - Jeg stoler ikke på helsepersonell

- *I do not trust healthcare professionals*

14 - Jeg stoler ikke på anbefaling fra helsemyndighetene/kommunen

- *I do not trust the recommendation from the health authorities / municipality*

15 – Mediaoppmerksomhet

- *Media attention*

16 - Annet

- *Different*

Q024 - Alder (fødselsdato)

Hva er din alder?

What is your age?

**open*

Later grouped into categories:

18-29 år - *18-29 years*

30-44 år - *30-44 years*

45-59 år - *45-59 years*

60+ år - *60+ years*

Q025 – Kjønn

Hva er ditt kjønn?

What is your gender?

1 - Kvinne – *Female*

2 - Mann - *Male*

Q026 – Utdanning

Hva er din høyeste fullførte skolegang?

What is your highest completed schooling?

1 - Grunnskoleutdanning - 10-årig grunnskole, 7-årig folkeskole

- *Primary school education - 10-year primary school, 7-year primary school*

2 - Videregående allmennfaglig utdanning

- *Upper secondary general education*

3 - Videregående yrkesfaglig utdanning

- *Upper secondary vocational educatio*n

4 - Fagskole / Yrkesrettede utdanninger (1/2 - 2 år) som bygger på videregående yrkesfaglig utdanning

- *Vocational school / Vocational education (1/2 - 2 years) based on upper secondary vocational education*

5 - Universitets-/høgskoleutdanning med inntil 4 års varighet

- *University / college education with up to 4 years duration*

6 - Universitets-/høgskoleutdanning med mer enn 4 års varighet

- *University / college education with more than 4 years duration*

Q030 - Husstands inntekt

Omtrent hvor stor er din husstands samlede brutto årsinntekt (før skatt og fradrag)?

Approximately how large is your household's total gross annual income (before taxes and deductions)?

1- Under 200 000 kroner - *Less than 200 000*

2 - 200 000 - 399 999 kroner

3 - 400 000 - 599 999 kroner

4 - 600 000 - 799 999 kroner

5 - 800 000 - 999 999 kroner

6 - 1 000 000 - 1 199 000 kroner

7 - 1 200 000 - 1 399 000 kroner

8 - 1 400 000 kroner eller mer - *1 400 000 kroners or more*

9 - Ønsker ikke å svare - *Do not want to answer*

Q031 – Fodelandnorge

Er du født i Norge?

Are you born in Norway?

1 - Ja – *yes*

2 - Nei – *no*
